# Association between HDL-cholesterol subfractions and the risk of major adverse cardiac and cerebrovascular events in patients with acute myocardial infarction: a single-center retrospective observational study

**DOI:** 10.3389/fnut.2025.1728525

**Published:** 2026-01-08

**Authors:** Yu Geng, Xingfei Deng, Yajun Xue, Bin Wang, Qing Liu, Tingting Lv, Changhua Lv, Yifei Wang, Ping Zhang

**Affiliations:** 1 Department of Cardiology, Beijing Tsinghua Changgung Hospital, School of Clinical Medicine, Tsinghua University, Beijing, China; 2 Ardent BioMed LLC (ABIOM), Guangzhou, Guangdong, China

**Keywords:** HDL subfractions, HDL-3, acute myocardial infarction, major adverse cardiac and cerebrovascular events, prognosis

## Abstract

**Background:**

This study aimed to evaluate the association between high-density lipoprotein (HDL) subfractions and long-term major adverse cardiac and cerebrovascular events (MACCEs) in patients with acute myocardial infarction (AMI).

**Method:**

A total of 1,240 AMI patients admitted to Beijing Tsinghua Changgung Hospital between 2017 and 2023 were included. HDL subfractions, including HDL-2b and HDL-3, were quantified using microfluidic chip electrophoresis. Patients were stratified into tertiles according to HDL-3 levels. The primary endpoint was the occurrence of MACCEs. Kaplan–Meier analysis, Cox proportional hazards models, restricted cubic spline (RCS), and mediation analyses were performed to evaluate the associations between HDL subfractions and MACCEs.

**Result:**

During a median follow-up of 52.4 months, 132 MACCEs (10.7%) occurred. Patients in the highest HDL-3 tertile had a lower MACCEs incidence than those in the lowest tertile (6.7% vs. 16.4%, *p* < 0.001). Higher HDL-3 levels were associated with improved event-free survival (HR = 0.58, 95% CI: 0.37–0.91) and demonstrated discriminative ability for MACCE risk (AUC = 0.62; 95% CI: 0.57–0.67), whereas HDL-C and HDL-2b were not significant. RCS analysis revealed a linear inverse association between HDL-3 and MACCEs (p for non-linearity = 0.356). The Gensini score partially mediated this relationship, accounting for 11.8% of the total effect.

**Conclusion:**

Lower HDL-3 levels were independently associated with a higher long-term risk of MACCEs in AMI patients. HDL-3 may represent a potential biomarker for residual cardiovascular risk stratification and a potential therapeutic target for improving post-infarction outcomes.

## 1 Introduction

Cardiovascular disease remains the leading cause of death worldwide, with ischemic heart disease (IHD) representing the dominant contributor, accounting for 8.99 million deaths in 2021 (1). Among the modifiable risk factors, metabolic disturbances are major determinants of IHD, responsible for 46.21% of IHD-related deaths in 2021 (2). Over the past three decades, the global burden attributable to metabolic risk factors has increased by 1.6 to 3 times (3), highlighting their significance as key therapeutic targets in cardiovascular prevention.

Lipid-lowering therapy, particularly statin treatment, remains the cornerstone for reducing low-density lipoprotein cholesterol (LDL-C) and preventing major adverse cardiac and cerebrovascular events (MACCEs) (4, 5). However, despite the substantial benefits of LDL-C reduction, a considerable residual cardiovascular risk persists (6). This persistent risk has renewed attention on high-density lipoprotein cholesterol (HDL-C), traditionally regarded as “good cholesterol” owing to its potential protective effects against atherosclerosis (7).

Nevertheless, accumulating evidence has challenged this traditional paradigm. Several cohort studies have reported a U-shaped association between HDL-C and both all-cause and cardiovascular mortality among patients with IHD (8), while randomized controlled trials aimed at pharmacologically increasing HDL-C levels failed to show reductions in cardiovascular events (9). These inconsistencies suggest that HDL-C functionality, rather than total HDL-C concentration, may better reflect cardiovascular protection.

HDL particles are highly heterogeneous, comprising distinct subfractions that differ in density, diameter, and biological function (10, 11). Large, cholesterol-rich HDL-2 particles (HDL-2a, HDL-2b) primarily facilitate reverse cholesterol transport, whereas small, dense HDL-3 particles (HDL-3a, HDL-3b, and HDL-3c) exhibit potent antioxidative and anti-inflammatory effects (12, 13). Previous studies have demonstrated that small HDL particles are inversely associated with coronary disease severity and mortality among patients with IHD (14–16).

Acute myocardial infarction (AMI), a major clinical manifestation of IHD, continues to be associated with substantial risks of recurrent events and long-term mortality despite advances in reperfusion therapy and secondary prevention (17, 18). However, evidence directly linking HDL subfractions to long-term outcomes in AMI remains limited. Moreover, variability in HDL subfraction measurement techniques across studies may contribute to heterogeneous findings. Therefore, this study used microfluidic chip electrophoresis to precisely quantify HDL subfractions (HDL-2b and HDL-3) and to evaluate their association with long-term MACCE risk in patients with AMI.

## 2 Method

### 2.1 Study population

This retrospective observational study included patients diagnosed with AMI who were admitted to Beijing Tsinghua Changgung Hospital between September 2017 and June 2023. Both ST-elevation myocardial infarction and non-ST-elevation myocardial infarction were eligible for inclusion. Patients were excluded if they met any of the following criteria: (1) missing serum samples for HDL subfraction measurement; (2) missing blood cholesterol data; (3) presence of active infection, autoimmune disease, severe hepatic or renal insufficiency, or malignancy; and (4) incomplete follow-up information.

**Figure 1:**
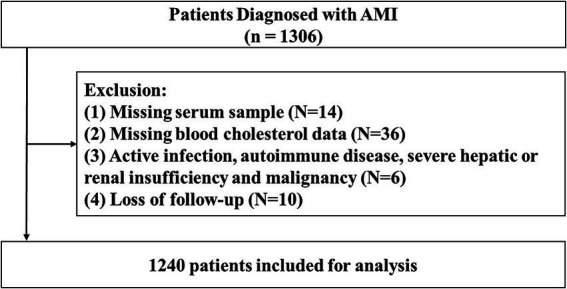
Flowchart of patient inclusion and exclusion criteria.

A total of 1,240 patients were included in the final analysis (Figure 1). The study protocol was conducted in accordance with the principles of the Declaration of Helsinki and was approved by the Ethics Committee of Beijing Tsinghua Changgung Hospital (Approval No. 23578-4-01). Given the retrospective design, the requirement for written informed consent was waived.

### 2.2 Measurement of HDL subfractions

Fasting venous blood samples were collected within 24 h of hospital admission. Serum was separated and stored at −80 °C until analysis. HDL subfractions were quantified using a microfluidic chip capillary electrophoresis system (MICEP-30, Ardent BioMed, Guangzhou, China) in accordance with the manufacturer’s instructions (19). Briefly, this technique separates HDL particles based on size and electrical charge, allowing quantification of HDL-2b and HDL-3 subfractions as percentages (%) of total HDL particles, from which corresponding concentrations were calculated. The intra-assay and inter-assay coefficients of variation were both <5%. Conventional lipid parameters, including total cholesterol (TC), LDL-C, and HDL-C, were measured using standard enzymatic methods in the central laboratory of Beijing Tsinghua Changgung Hospital.

### 2.3 Clinical data and definitions

Demographic characteristics, comorbidities, laboratory data, and coronary angiographic findings were extracted from the hospital’s electronic health record system. Hypertension was defined as systolic blood pressure ≥140 mmHg, diastolic blood pressure ≥90 mmHg, or current use of antihypertensive drugs. Diabetes mellitus was defined as fasting glucose ≥7.0 mmol/L, HbA1c ≥ 6.5%, or current use of antidiabetic therapy. Chronic kidney disease (CKD) was defined as an estimated glomerular filtration rate (eGFR) < 60 mL/min/1.73 m^2^. Statin use was recorded if the patient was prescribed statin therapy at discharge. The severity of coronary artery stenosis was assessed using the Gensini score. Coronary angiograms were independently evaluated by two cardiologists who were blinded to patient outcomes, and discrepancies were resolved by consensus.

### 2.4 Outcomes and follow-up

The primary endpoint was the occurrence of MACCEs, defined as a composite of all-cause death, recurrent myocardial infarction (Re-MI), stroke, and major bleeding events (intracranial hemorrhage or gastrointestinal tract bleeding).

Follow-up information was obtained through outpatient visits, hospital records, and structured telephone interviews conducted every 6–12 months. The follow-up period was calculated from the date of the index AMI admission to the date of the first MACCE event or the last available contact.

### 2.5 Statistical analysis

Continuous variables were expressed as medians (Q1, Q3), and categorical variables were presented as counts (percentages). Differences among HDL subfraction tertile groups were compared using the Kruskal–Wallis test for continuous variables and the *χ*^2^ test for categorical variables.

Receiver operating characteristic (ROC) curve analysis was conducted to evaluate the discriminative ability of HDL-C, HDL-2b, and HDL-3 for predicting MACCEs. The area under the curve (AUC) and 95% confidence intervals (CIs) were calculated using DeLong’s non-parametric method, and AUCs were compared across HDL subfractions. Event-free survival was evaluated with Kaplan–Meier curves, and between-group differences were assessed with the log-rank test.

To examine the association between HDL subfractions and MACCEs, Cox proportional hazards regression models were constructed, and hazard ratios (HRs) with 95% CIs were estimated. HDL subfractions were analyzed both as tertiles and as continuous variables (per 0.1 mmol/L increment). Three hierarchical models were fitted, and the proportional hazards assumption was tested using Schoenfeld residuals and was satisfied for all models:

- Model 1: unadjusted;
- Model 2: adjusted for age and sex;
- Model 3: further adjusted for hypertension, diabetes mellitus, CKD, and statin use. Restricted cubic spline (RCS) models with four knots were applied to evaluate potential non-linear relationships between HDL subfractions and MACCEs risk. To explore potential mechanisms, mediation analysis was performed to assess whether the Gensini score mediated the association between HDL-3 and MACCEs, using a bias-corrected bootstrap approach with 1,000 replications. Subgroup analyses were further conducted according to age, sex, hypertension, diabetes, and CKD status, and multiplicative interaction terms were included to test for effect modification.

All analyses were performed using R software (version 4.4.3), with two-tailed *p* < 0.05 considered statistically significant.

## 3 Results

### 3.1 Baseline characteristics

Among the 1,240 patients included in this study, the median age was 64 years, and 25.1% were women (Table 1). Patients were stratified into three groups according to HDL-3 tertiles for comparison. Individuals in the lowest HDL-3 tertile were significantly older (*p* = 0.001) and had a higher prevalence of CKD (*p* < 0.001). In contrast, the distributions of sex, hypertension, and diabetes mellitus were comparable across HDL-3 tertiles. In addition, patients with lower HDL-3 levels had substantially reduced eGFR values (*p* < 0.001) and elevated C-reactive protein levels (*p* < 0.001), suggesting more pronounced renal dysfunction and systemic inflammation.

**Table 1:** Baseline characteristics of the study population stratified by HDL-3 tertiles.

Variables	Total (*n* = 1,240)	Low (*n* = 409)	Medium (*n* = 422)	High (*n* = 409)	*P*
Age, years	64.00 (55.75, 72.00)	65.00 (56.00, 76.00)	62.00 (55.00, 71.00)	63.00 (54.00, 71.00)	0.001
Age category, years					0.018
<45	112 (9.03)	34 (8.31)	44 (10.43)	34 (8.31)	
45–60	555 (44.76)	159 (38.88)	200 (47.39)	196 (47.92)	
≥60	573 (46.21)	216 (52.81)	178 (42.18)	179 (43.77)	
Female	311 (25.08)	89 (21.76)	116 (27.49)	106 (25.92)	0.146
Hypertension	869 (70.08)	285 (69.68)	291 (68.96)	293 (71.64)	0.685
Diabetes mellitus	525 (42.34)	184 (44.99)	177 (41.94)	164 (40.10)	0.360
CKD	214 (17.26)	98 (23.96)	58 (13.74)	58 (14.18)	<0.001
Statin use	1,130 (91.13)	367 (89.73)	391 (92.65)	372 (90.95)	0.330
HDL, mmol/L	0.91 (0.77, 1.08)	0.78 (0.67, 0.96)	0.87 (0.77, 1.00)	1.03 (0.91, 1.21)	<0.001
HDL2b, mmol/L	0.20 (0.15, 0.27)	0.18 (0.13, 0.25)	0.20 (0.15, 0.26)	0.23 (0.18, 0.31)	<0.001
HDL3, mmol/L	0.31 (0.25, 0.38)	0.22 (0.17, 0.25)	0.31 (0.29, 0.33)	0.40 (0.38, 0.45)	<0.001
CRP, mg/L	4.63 (1.77, 10.30)	7.17 (3.00, 20.59)	4.15 (1.89, 9.07)	3.13 (1.40, 6.88)	<0.001
TC, mmol/L	4.31 (3.58, 5.05)	3.92 (3.33, 4.66)	4.34 (3.65, 5.00)	4.66 (3.90, 5.37)	<0.001
LDL, mmol/L	2.72 (2.08, 3.44)	2.47 (1.90, 3.07)	2.78 (2.13, 3.47)	2.97 (2.25, 3.71)	<0.001
PLT, 10^9/L	215 (176, 252)	203 (168, 247)	223.50 (189, 255)	214 (182, 254)	<0.001
HbA1c, %	6.20 (5.70, 7.60)	6.20 (5.60, 7.65)	6.25 (5.70, 7.60)	6.20 (5.70, 7.40)	0.830
eGFR, mL/min/1.73 m^2^	91.76 (71.97, 102.80)	87.05 (62.85, 101.13)	93.91 (78.48, 103.89)	92.72 (74.09, 103.16)	<0.001
Gensini score	52.00 (28.00, 82.38)	58.00 (32.62, 90.00)	52.00 (28.00, 80.00)	48.00 (25.00, 80.00)	0.002
MACCE, *n* (%)	132 (10.65)	67 (16.38)	38 (9.00)	27 (6.60)	<0.001
Death, *n* (%)	42 (3.39)	22 (5.38)	11 (2.61)	9 (2.20)	0.023
Re-MI, *n* (%)	57 (4.60)	29 (7.09)	17 (4.03)	11 (2.69)	0.009
Stroke, *n* (%)	21 (1.69)	11 (2.69)	6 (1.42)	4 (0.98)	0.144
Bleeding, *n* (%)	12 (0.97)	5 (1.22)	4 (0.95)	3 (0.73)	0.826

CKD, chronic kidney disease; CRP, C-reactive protein; eGFR, estimated glomerular filtration rate; HbA1c, glycated hemoglobin; HDL, high-density lipoprotein; LDL, low-density lipoprotein; MACCE, major adverse cardiac and cerebrovascular events; PLT, platelet count; Re-MI, recurrent myocardial infarction; TC, total cholesterol.

Data are presented as median (Q1, Q3) for continuous variables and number (percentage) for categorical variables.

### 3.2 Association between HDL subfractions and MACCEs risk

During a median follow-up of 52.4 months, 132 patients (10.7%) experienced MACCEs, including 42 deaths (3.4%) and 57 recurrent myocardial infarctions (4.6%) (Table 1).

**Figure 2:**
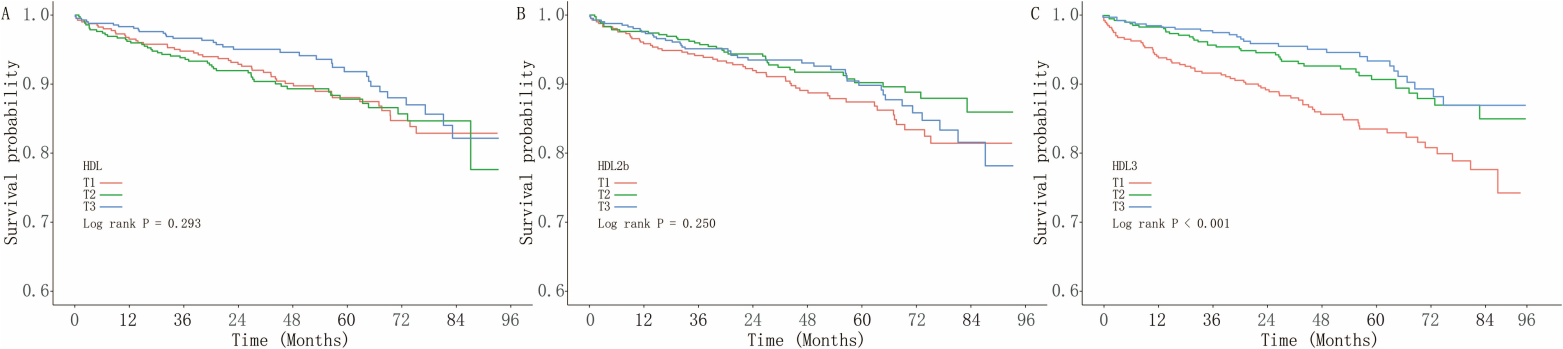
Kaplan–Meier survival curves for MACCEs by HDL subfraction tertiles. Kaplan–Meier survival curves showing the cumulative incidence of MACCEs across tertiles of **(A)** total HDL-C, **(B)** HDL-2b, and **(C)** HDL-3. Differences among tertile groups were evaluated using the log-rank test.

The Kaplan–Meier analysis showed no significant differences in MACCEs risk across tertiles of total HDL-C or HDL-2b (log-rank *p* > 0.05; Figures 2A,B). In contrast, the cumulative incidence of MACCEs decreased progressively across increasing HDL-3 tertiles (log-rank *p* < 0.001; Figure 2C).

**Figure 3:**
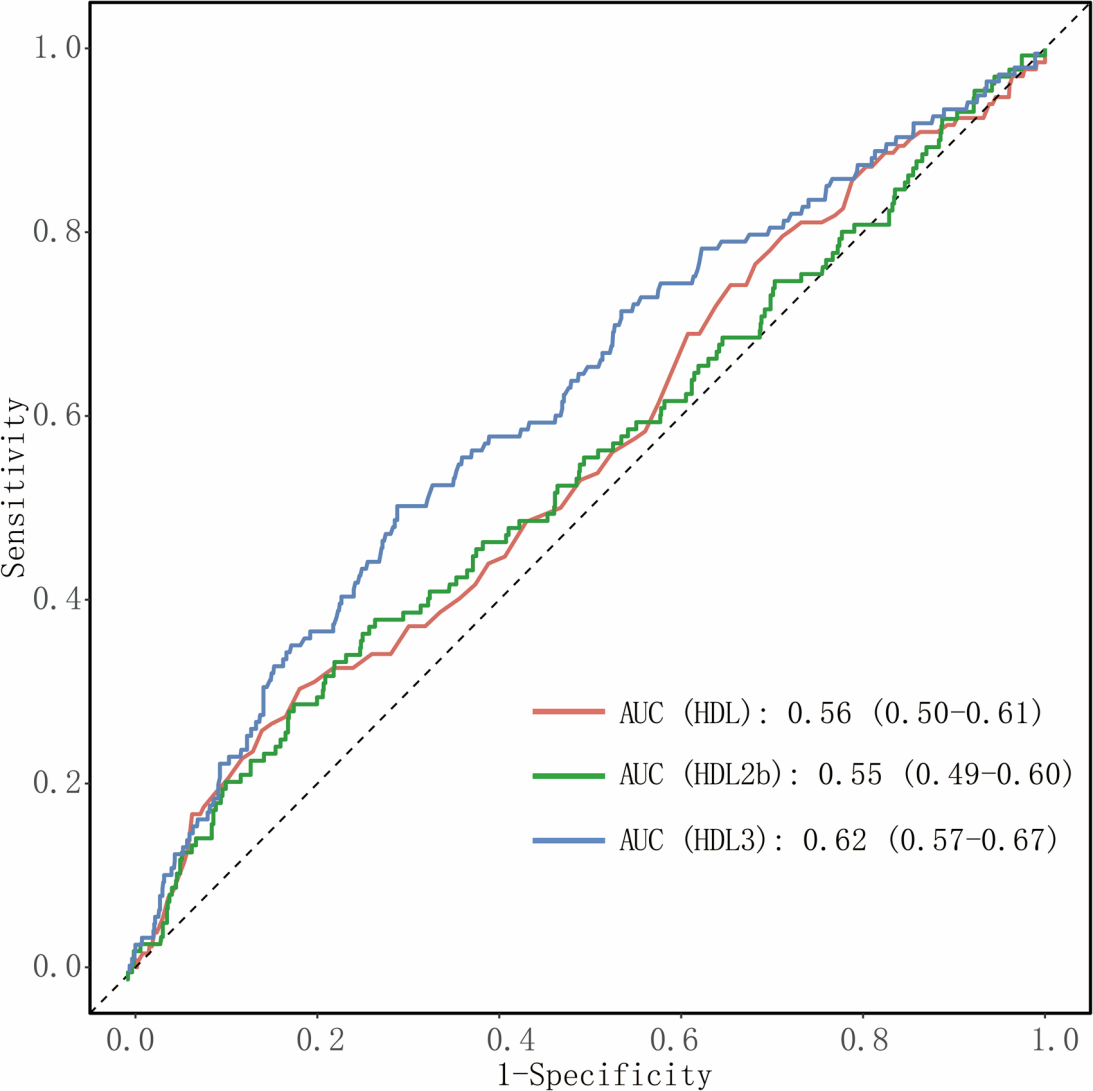
ROC curves of HDL subfractions for predicting MACCEs. ROC curves illustrating the discriminative performance of total HDL-C, HDL-2b, and HDL-3 for predicting long-term MACCEs in patients with AMI.

ROC analysis showed that HDL-C (AUC = 0.56; 95% CI: 0.50–0.61) and HDL-2b (AUC = 0.55; 95% CI: 0.49–0.60) showed limited discriminative ability for predicting MACCEs (Figure 3). In comparison, HDL-3 demonstrated stronger predictive performance (AUC = 0.62; 95% CI: 0.57–0.67).

In univariate Cox regression analysis, neither higher HDL-C nor HDL-2b levels were significantly associated with MACCEs risk compared with the lowest tertile (*p* > 0.05). In contrast, higher HDL-3 levels were associated with a lower MACCEs risk (Table 2). The HRs for MACCEs across HDL-3 tertiles were 0.56 (95% CI: 0.37–0.83) for the middle tertile and 0.43 (95% CI: 0.28–0.68) for the highest tertile. When analyzed as a continuous variable, each 0.1 mmol/L increment in HDL-3 was associated with a 28% reduction in MACCEs risk (HR = 0.72; 95% CI: 0.61–0.85).

**Table 2:** Cox regression models for the association between HDL subfractions and MACCEs risk.

Variables	Model 1		Model 2		Model 3	
	HR (95% CI)	*P*	HR (95% CI)	*P*	HR (95% CI)	*P*
HDL						
Low	Reference		Reference		Reference	
Medium	1.04 (0.70–1.54)	0.861	0.95 (0.64–1.41)	0.796	1.03 (0.69–1.54)	0.888
High	0.75 (0.48–1.16)	0.193	0.57 (0.36–0.89)	0.014	0.64 (0.40–1.02)	0.060
*P* for trend		0.212		0.015		0.066
Per 0.1 mmol/L increase	0.97 (0.90–1.04)	0.357	0.93 (0.86–0.99)	0.043	0.95 (0.88–1.02)	0.151
HDL-2b						
Low	Reference		Reference		Reference	
Medium	0.71 (0.46–1.07)	0.100	0.61 (0.40–0.93)	0.023	0.68 (0.45–1.04)	0.078
High	0.83 (0.55–1.25)	0.370	0.58 (0.38–0.88)	0.010	0.65 (0.43–1.00)	0.050
*P* for trend		0.336		0.010		0.047
Per 0.1 mmol/L increase	0.92 (0.78–1.10)	0.373	0.81 (0.68–0.97)	0.020	0.86 (0.72–1.03)	0.093
HDL-3						
Low	Reference		Reference		Reference	
Medium	0.56 (0.37–0.83)	0.004	0.62 (0.42–0.93)	0.022	0.76 (0.50–1.14)	0.186
High	0.43 (0.28–0.68)	<0.001	0.48 (0.31–0.75)	0.001	0.58 (0.37–0.91)	0.019
*P* for trend		<0.001		<0.001		0.016
Per 0.1 mmol/L increase	0.72 (0.61–0.85)	<0.001	0.76 (0.65–0.90)	0.001	0.83 (0.70–0.98)	0.028

HR with 95% CIs were estimated using both tertile-based and continuous models, with the lowest tertile serving as the reference category. HRs for each 0.1 mmol/L increment were additionally calculated.

Model 1: unadjusted.

Model 2: adjusted for age and sex.

Model 3: adjusted for age, sex, hypertension, diabetes mellitus, CKD, and statin use.

The inverse association between HDL-3 and MACCEs remained significant after adjusting for potential confounding factors (Table 2). In the fully adjusted model (Model 3: adjusted for age, sex, hypertension, diabetes, CKD, and statin use), patients in the highest HDL-3 tertile had a significantly lower risk of MACCEs compared with those in the lowest tertile (HR = 0.58; 95% CI: 0.37–0.91). In the continuous model, each 0.1 mmol/L increase in HDL-3 was associated with a 17% reduction in the risk of MACCEs (HR = 0.83; 95% CI: 0.70–0.98).

### 3.3 Linear relationship between HDL-3 and MACCEs

**Figure 4:**
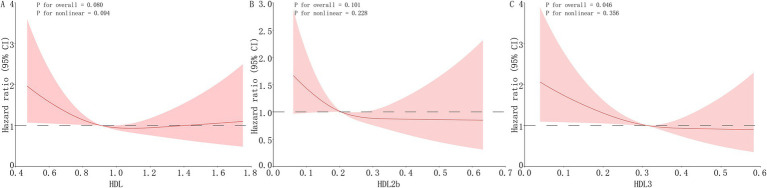
RCS analyses for the association between HDL subfractions and MACCEs. RCS analyses illustrating the dose–response relationships between HDL subfractions and MACCEs risk. Models were adjusted for all covariates included in Model 3 (age, sex, hypertension, diabetes mellitus, chronic kidney disease, and statin use). Panels show **(A)** total HDL-C, **(B)** HDL-2b, and **(C)** HDL-3.

RCS models adjusted for all covariates in Model 3 were used to evaluate the dose–response relationships between HDL subfractions and MACCEs risk. Neither HDL-C nor HDL-2b showed a significant association with MACCEs (*p* for overall > 0.05; Figures 4A, B). In contrast, HDL-3 demonstrated a linear inverse association with MACCEs (*p* for non-linearity = 0.356; Figure 4C).

### 3.4 Mediation and subgroup analyses

**Figure 5:**
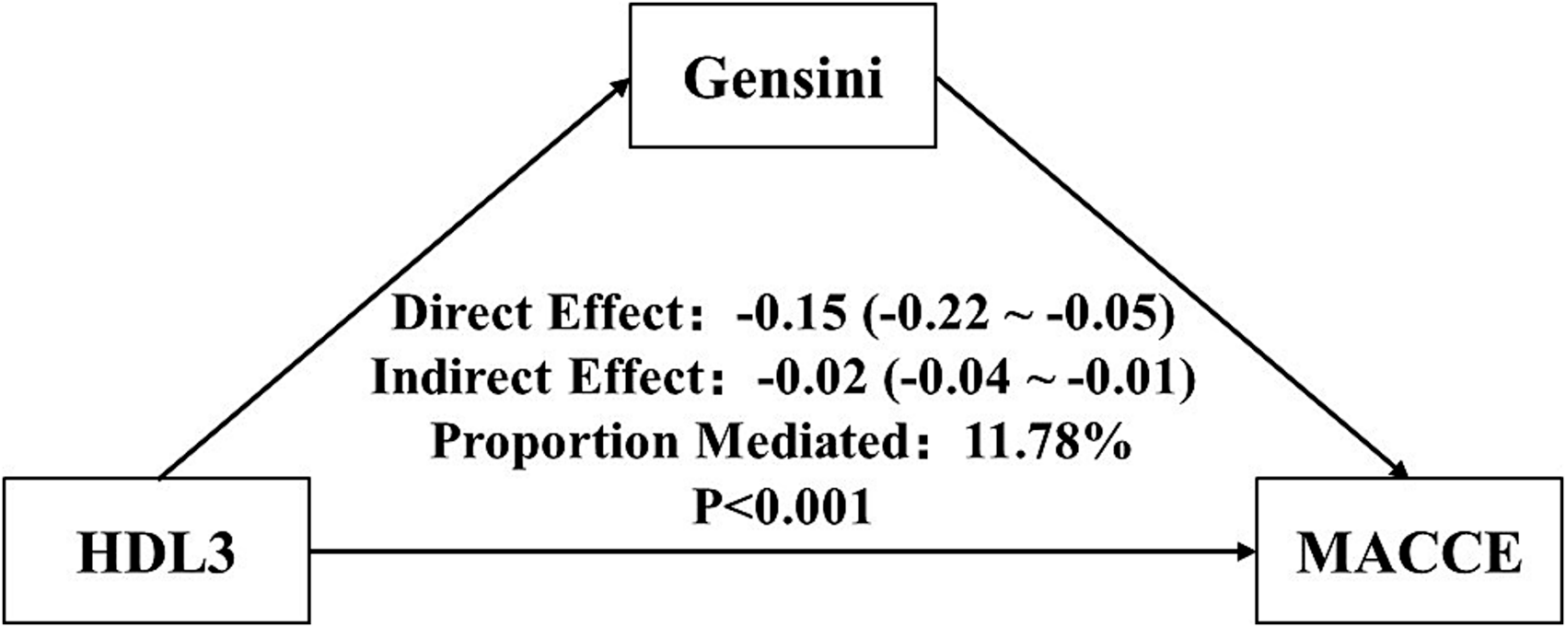
Mediation analysis of the association between HDL-3 and MACCEs. Mediation analysis evaluating the indirect effect of the Gensini score on the association between HDL-3 and MACCEs.

Mediation analysis indicated that the Gensini score partially mediated the association between HDL-3 and MACCEs. Higher HDL-3 levels were inversely associated with the Gensini score, and this mediation pathway accounted for 11.8% of the overall protective effect of HDL-3 on MACCEs risk (Figure 5).

Subgroup analyses were performed to assess potential effect modification by demographic factors (age and sex) and comorbidities (hypertension, diabetes mellitus, and CKD). The inverse association between HDL-3 and MACCEs was consistent across age, sex, hypertension, and CKD subgroups (all *p* for interaction > 0.05). Notably, the protective association was stronger among patients with diabetes mellitus (*p* for interaction = 0.001; Table 3).

**Table 3:** Subgroup analyses of the association between HDL-3 and MACCEs risk.

Variables	HR (95% CI)	*P*	*P* for interaction
Age			0.504
<45	0.39 (0.11–1.38)	0.144	
45–60	0.70 (0.49–1.01)	0.058	
≥60	0.80 (0.67–0.97)	0.022	
Sex			0.799
Male	0.75 (0.61–0.93)	0.009	
Female	0.82 (0.63–1.06)	0.127	
Hypertension			0.712
No	0.86 (0.58–1.27)	0.453	
Yes	0.76 (0.64–0.92)	0.004	
Diabetes mellitus			0.001
No	1.05 (0.82–1.34)	0.705	
Yes	0.60 (0.48–0.75)	<0.001	
CKD			0.681
No	0.86 (0.69–1.07)	0.169	
Yes	0.80 (0.61–1.05)	0.105	

Cox proportional hazards models examining the association between HDL-3 levels and MACCEs across subgroups defined by age, sex, hypertension, diabetes mellitus, and CKD.

## 4 Discussion

In this single-center longitudinal cohort of patients with AMI, lower levels of the HDL-3 subfraction were significantly associated with a higher risk of long-term MACCEs, whereas total HDL-C and HDL-2b showed no independent predictive value. The inverse association between HDL-3 and MACCEs was linear and was partially mediated by the severity of coronary atherosclerosis, as quantified by the Gensini score. Notably, the protective effect of HDL-3 was most pronounced in patients with diabetes mellitus.

These results extend previous cross-sectional findings by demonstrating the long-term prognostic relevance of HDL subfractions in AMI patients. Earlier studies have reported that both HDL-2b and HDL-3 are inversely correlated with the severity of coronary stenosis, whereas total HDL-C did not display a consistent pattern (15, 20). Additional clinical studies across diverse cardiovascular populations also support the prognostic value of HDL-3. For instance, Kim et al. observed that HDL-3 exhibited a stronger protective association than HDL-2 in patients with carotid artery disease (21). Similarly, among individuals with heart failure, HDL-3, but not HDL-2 or total HDL-C, was significantly associated with reduced 3-month mortality (22). In two independent AMI cohorts (*n* = 2,465 and 2,414, respectively), HDL-3 was inversely associated with long-term adverse cardiac events (23). Furthermore, several studies focusing on HDL particle size have emphasized that smaller HDL particles provide better predictive power for mortality among patients with cardiovascular disease (24, 25).

HDL-3 represents the small, dense HDL particles enriched with functional proteins such as paraoxonase-1 and apolipoprotein A-I, which confer potent antioxidative and anti-inflammatory properties (26–28). Mechanistically, HDL-3, often described as small-sized HDL particles, can inhibit low-density lipoprotein oxidation, reduce endothelial adhesion molecule expression, and promote cholesterol efflux via ATP-binding cassette transporter A1 (ABCA1) (29–31). These biological effects limit macrophage foam-cell formation, suppress vascular inflammation within the arterial wall, and stabilize vulnerable plaques, thereby reducing the likelihood of recurrent ischemic events (32, 33). The mediation effect of the Gensini score observed in our study supports these classical anti-atherogenic pathways, suggesting that higher HDL-3 levels may help reduce atherosclerotic burden and promote coronary stability.

Nevertheless, mediation analysis showed that only 11.8% of the association between HDL-3 and clinical outcomes was explained by the Gensini score, implying that the majority of HDL-3’s protective effects are mediated through non-atherosclerotic pathways. Beyond plaque-related pathways, HDL-3 exerts systemic anti-inflammatory and antioxidative effects beyond the arterial plaque. By scavenging reactive oxygen species, modulating toll-like receptor signaling, and suppressing NLRP3 inflammasome activation, HDL-3 dampens post-infarction sterile inflammation and ventricular remodeling independent of plaque burden (34–37). In addition, HDL can improve endothelial function and microvascular perfusion via a sphingosine-1-phosphate-dependent pathway, further enhancing prognosis. More importantly, sphingosine-1-phosphate has been reported to be preferentially enriched in small, dense HDL3 particles (38, 39). Additionally, HDL-3 demonstrates antithrombotic and antiplatelet properties, reducing platelet aggregation and tissue-factor expression while enhancing endothelial prostacyclin and nitric oxide release, thereby decreasing the likelihood of thrombosis-driven recurrent events (40, 41). Collectively, these pleiotropic functions, including anti-inflammatory, vasodilatory, and antithrombotic effects, provide a biologically plausible explanation for the prognostic benefit of HDL-3 that is not mediated by coronary atherosclerosis.

A notable finding from this study is the significant interaction between HDL-3 and diabetes mellitus in determining the long-term prognosis of patients with AMI. Previous studies have reported associations between HDL subfractions and metabolic syndrome in individuals with premature coronary heart disease (42, 43). In diabetes, chronic hyperglycemia leads to oxidative modification and non-enzymatic glycation of HDL-associated proteins, including apolipoprotein A-I and paraoxonase-1, resulting in impaired cholesterol efflux capacity, reduced antioxidative activity, and a shift toward a pro-inflammatory HDL phenotype. Recent reviews further highlight that these compositional and enzymatic alterations contribute to the formation of dysfunctional HDL, characterized by reduced capacity to suppress vascular inflammation and preserve endothelial function (44). The stronger protective effect of HDL-3 observed in diabetic patients in our study may therefore reflect that, in a metabolically stressed environment, individuals with relatively preserved HDL-3 functionality derive the greatest cardiovascular benefit. In contrast, among non-diabetic individuals, who generally exhibit lower oxidative and inflammatory burden and experience fewer events, the functional gradient across HDL subfractions may be less pronounced, potentially explaining the absence of a significant association in this subgroup.

The findings of this study suggest that HDL subfractions, particularly the small, dense HDL-3 particles, may serve as practical and sensitive biomarkers for risk stratification in patients after AMI. Unlike total HDL-C, which represents a heterogeneous assemblage of HDL particles differing in size and function, HDL-3 directly reflects the most bioactive fraction with antioxidative and anti-inflammatory potential (33). Incorporating HDL-3 measurement into routine lipid profiling may help identify high-risk patients with elevated residual risk who could benefit from intensified lipid-lowering or anti-inflammatory therapies.

Several limitations of this study should be acknowledged. First, as an observational single-center cohort, causal relationships cannot be established, and unmeasured confounding factors may still exist despite multivariable adjustment. Second, HDL-3 was measured only at baseline, and longitudinal changes in HDL subfractions during follow-up were not assessed. Third, information on non-statin lipid-lowering therapies was not included because their prescription rates were extremely low, although a residual effect cannot be fully excluded. Finally, external validation in multicenter, prospective cohorts is needed to confirm the generalizability of these findings.

## 5 Conclusion

In this retrospective cohort of patients with AMI, lower HDL-3 subfraction levels were independently and linearly inversely associated with a higher long-term risk of MACCEs, whereas total HDL-C and HDL-2b did not demonstrate significant prognostic value. Part of the association between HDL-3 and cardiovascular outcomes was mediated by the severity of coronary atherosclerosis, as reflected by the Gensini score. These findings suggest that HDL-3 represents a functionally relevant component of HDL metabolism and may more accurately reflect residual cardiovascular risk than conventional lipid parameters.

## Data Availability

The raw data supporting the conclusions of this article will be made available by the authors, without undue reservation.
